# A Case of CIED-Associated Endocarditis and Septic Emboli Requiring Lead Extraction, AngioVac Suction, and Pulmonary Endarterectomy

**DOI:** 10.7759/cureus.11601

**Published:** 2020-11-20

**Authors:** Erik A Green, Travis Pollema, Victor Pretorius

**Affiliations:** 1 Surgery, Tulane University School of Medicine, New Orleans, USA; 2 Cardiothoracic Surgery, University of California San Diego School of Medicine, La Jolla, USA

**Keywords:** device-associated endocarditis, septic emboli, lead extraction, angiovac, pulmonary thromboendarterectomy

## Abstract

Cardiac Implantable Electronic Devices (CIED)-associated infective endocarditis complicated by septic emboli and acute on chronic pulmonary hypertension is rare. We present a case where pulmonary thromboendarterectomy was required for treatment. A 55 year-old man with a history of myocardial infarction and ischemic cardiomyopathy status-post ICD placement 8 years prior presented with bacteremia, infected ICD, and tricuspid valve vegetation. He underwent CIED extraction along with the use of the AngioVac suction device to remove right ventricular and atrial vegetations. However the patient had persistent valvular vegetation and bilateral sub-massive pulmonary emboli. Pulmonary angiography showed filling defects in the lobar and segmental arteries. Percutaneous attempts at embolectomy were unsuccessful and he therefore underwent a pulmonary endarterectomy surgery (PTE).

This case of CIED- associated endocarditis demonstrates the importance of early aggressive treatment of such infections. Guidelines recommend compete CIED system removal when there is associated infection. The AngioVac is a novel system for removal of right-sided vegetations and thrombi; however, complications such as distal embolization can occur. PTE surgery for septic emboli is rare. However, cases of such treatment as is presented here can be successful and may be necessary should percutaneous methods fail.

## Introduction

Cardiac Implantable Electronic Devices (CIED)-associated infective endocarditis complicated by septic emboli and acute on chronic pulmonary hypertension is rare. Here we present a case where pulmonary thromboendarterectomy (PTE) was required to successfully treat this uncommon complication.

## Case presentation

**Figure 1:**
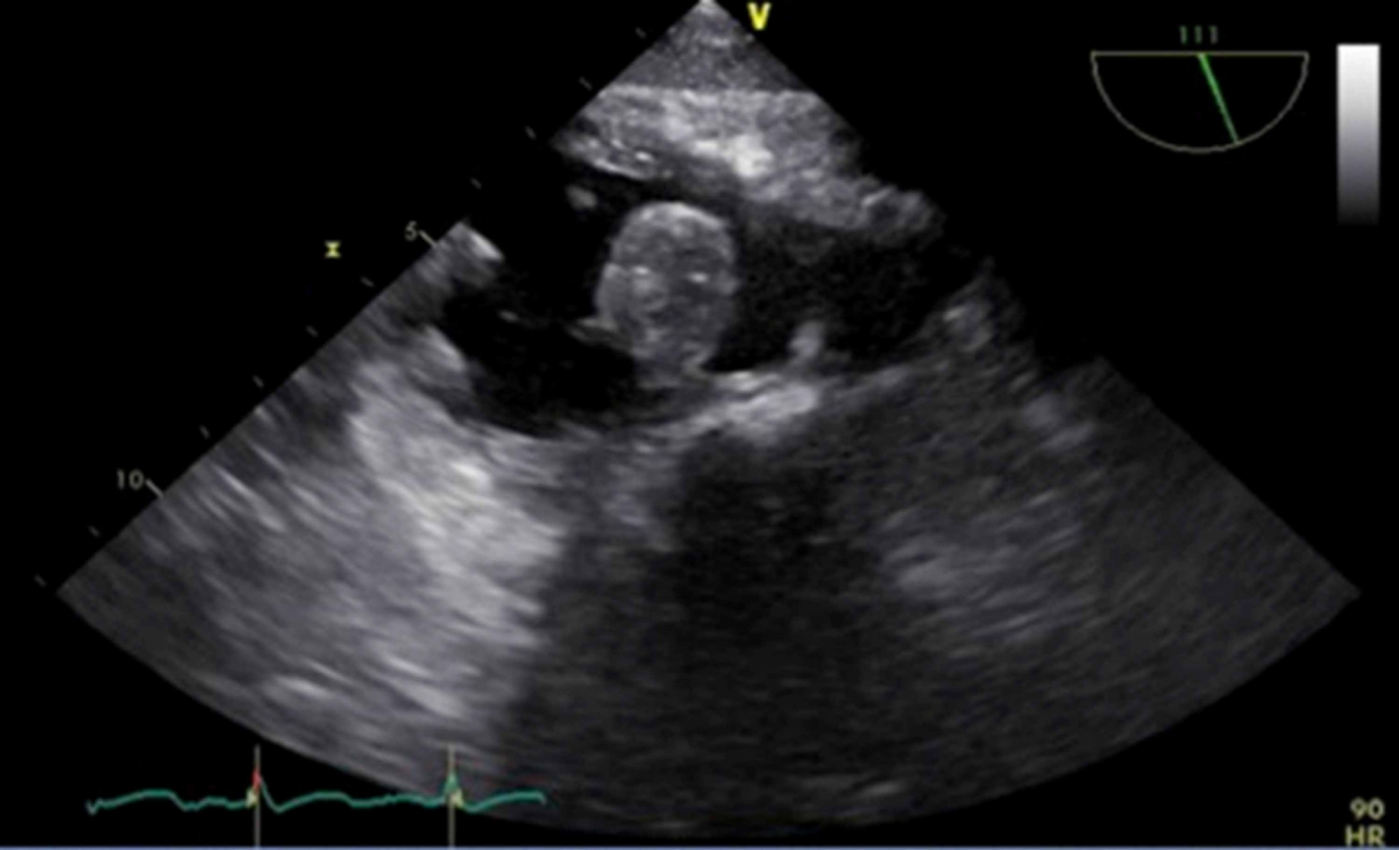
TEE imaging showing large mobile tricuspid valve vegetation associated with the CIED lead

A 55 year-old man with a history of myocardial infarction and ischemic cardiomyopathy status-post ICD placement 8 years prior presented to an outside hospital with bacteremia. His original ICD placement was in 2005, with a Guidant FineLine atrial lead and a Guidant dual coil ICD lead. In 2008, an additional Guidant atrial lead was placed. He was transferred to our tertiary care center after 8 weeks of IV antibiotics and evidence of a large 5cm x 2cm, mobile tricuspid valve vegetation associated with the ICD lead (Figure 1) seen on trans-Esophageal Echocardiogram (TEE). Blood cultures were positive for Staphylococcus hominis.

**Figure 2:**
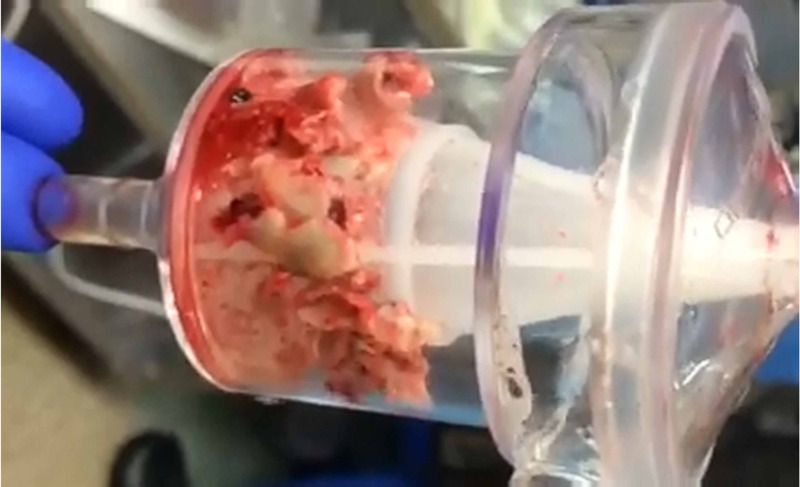
RA vegetation and debris post AngioVac suction

The patient was taken to the hybrid OR, placed under general anesthesia, and underwent pulse generator and lead extraction, along with AngioVac removal of RV and RA vegetations (Figure 2). Significant purulent fluid was found in the device pocket and tracking along the leads. The RA FineLine lead was extracted with a 14F laser sheath. However, adhesions made extraction difficult and the lead tip was fractured and was subsequently removed using a femoral approach with a snare. The AngioVac system was then used to remove the large vegetation via right internal jugular and right femoral vein cannulation. The patient was heparinized to an ACT >250. The AngioVac cannula was advanced into the RA, and 1.5-2L/min flow was achieved. The 14F laser sheath was used to extract the second atrial lead and a 16F laser sheath and a mechanical rotational tool was then required to extract the ICD lead.

Post-procedure the patient remained febrile and with leukocytosis to 23,000. Post-procedure TEE showed persistent valve vegetation and a chest CT scan showed evidence of sub-massive pulmonary emboli. The patient had worsening fevers, chills, pleuritic pain, and dyspnea despite antibiotics. Right heart catheterization revealed normal right heart pressures and borderline pulmonary hypertension with a mean pulmonary artery pressure of 27 mmHg. Pulmonary angiography showed proximal filling defects of bilateral lobar and segmental branches. Percutaneous embolectomy was attempted but was unsuccessful as a wire was unable to be passed beyond the thrombus.

**Figure 3:**
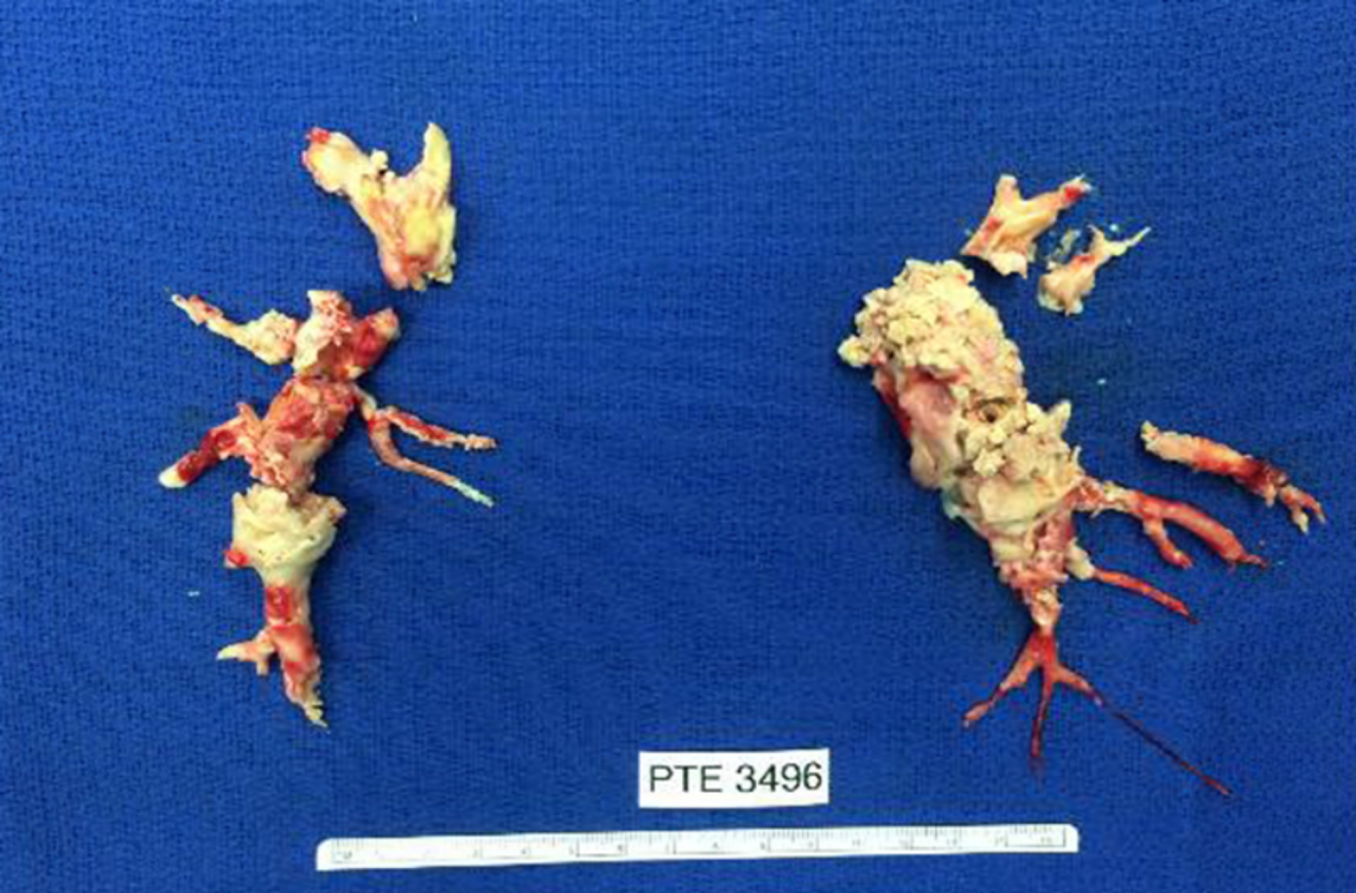
Left and Right Pulmonary Endarterectomy specimen

The patient was therefore considered for pulmonary endarterectomy surgery. He underwent bilateral pulmonary endarterectomy under cardiopulmonary bypass and deep hypothermic circulatory arrest (287 minutes of bypass time, 23 minutes of arrest time on right side, 20+18 minutes of arrest on left) (Figure 3). The tricuspid valve and right ventricle vegetations were also removed and the septal leaflet of the tricuspid valve repaired with a Gortex neochord. His post-operative course was uneventful with resolution of symptoms and improved hemodynamics with a Pulmonary Vascular Resistance of 146, down from 316 dyne*sec/cm5. He was discharged on antibiotics and 6 months of anticoagulation. He has done well in follow-up and a new ICD system was later successfully placed at his referring institution.

## Discussion

This case of CIED- associated endocarditis demonstrates the importance of early aggressive treatment of such infections and also highlights the importance of strategic lead management.

The 2017 Heart Rhythm Society (HRS) lead management guidelines [1] emphasize the importance of recognizing CIED and lead infection early and that quick implementation of appropriate antibiotic therapy should be initiated. Infection is one of the strongest indications for device removal. The HRS consensus issued a class I recommendation for complete CIED and lead removal within 3 days of infectious diagnosis [1]. Delay of removal, even with appropriate antibiotics, increases the risk of mortality and morbidity, as in this case when the patient came to extraction only after 8 weeks of antibiotic therapy. In CIED infection with associated vegetations, the HRS lead management guidelines similarly suggest device reimplantation after completion of appropriate therapy with 4-6 weeks of antibiotics, depending on the microbiology, and confirmed negative blood cultures [1].

The AngioVac system is a relatively novel method for removal of material from the vasculature using veno-venous bypass. Studies have shown it to be an effective method of percutaneous removal of thrombi and right-sided vegetations [2-4]. Reports have also shown it to be useful in management of vegetations associated with pacemaker and defibrillator leads [5-6]. However, as in the patient presented here, cases of incomplete extraction and distal embolization have been reported. This may be more common with chronic or well-organized clots [7-8].

There are a few reports of PTE for septic emboli in the literature [9-10]; however, none associated with a CIED. These few include reports of successful outcomes as well as a report of two cases that were unsuccessful due to inadequate separation between the vessel walls and the embolic masses. In the case presented here, surgery was successful and the patient did well.

## Conclusions

In this case report, we demonstrate a successful treatment of a complicated case of CIED-associated infection and endocarditis. This patient did not undergo early device removal, and despite efforts at minimally invasive techniques of lead and vegetation removal, our patient eventually required an open surgery. This shows the importance of early antibiotic therapy to minimize the development and growth of vegetations, little delay in device removal, and close monitoring to determine when to proceed with an open extraction.
